# Acinic Cell Carcinoma of the Breast Arising in Microglandular Adenosis

**DOI:** 10.1155/2013/736048

**Published:** 2013-11-28

**Authors:** Jessica Falleti, Gino Coletti, Ettore Rispoli, Francesca Scarabeo, Mariarosaria Cervasio, Luigi Tornillo, Guido Pettinato, Luigi Insabato

**Affiliations:** ^1^Section of Pathology, Department of Advanced Biomedical Sciences, University “Federico II” Naples, Via Pansini 5, 80131 Naples, Italy; ^2^Pathology Department, F.Veneziale Hospital, 86170 Isernia, Italy; ^3^Pathology Department, S. Salvatore Hospital, 67100 L'Aquila, Italy; ^4^Breast Unit, F.Veneziale Hospital, 86170 Isernia, Italy; ^5^Institute of Pathology, University of Basel, 4003 Basel, Switzerland

## Abstract

Acinic cell carcinoma is a rare breast tumour belonging to salivary gland-like tumours of the breast. They are “triple-negative” breast cancers even if their biological behaviour seems to be more favourable. Herein we present an acinic cell carcinoma arising on a background of typical and atypical microglandular adenosis in a 58-year-old woman, along with a review of the literature.

## 1. Introduction

Acinic cell carcinoma (ACC) belongs to the group of salivary gland-like tumours of the breast.

This rare malignant tumour, first described in 1996 by Roncaroli et al. [1], is essentially the breast counterpart of a similar tumour commonly found in the parotid gland and showing a diffuse infiltrative growth pattern with small acinar or glandular structures which are composed of monotonous proliferation of cells with a granular or clear cytoplasm, thus resembling acinar cells of the salivary glands [2, 3].

This report presents a full description of a rare case of primary ACC of the breast arising in microglandular adenosis (MGA) in a 58-year-old woman which showed also a previous history of thyroid cancer.

We reviewed the literature on the subject.

To the best of our knowledge, among 26 reported cases of ACC [1, 4–18], we were able to disclose only one previous case [9].

## 2. Case Report

A 58-year-old woman, with a prior thyroid cancer history about 13 years ago, detected a firm nodular lesion in the periareolar region of her right breast self-examination.

Subsequently mammography and echographic studies were performed.

**Figure 1:**
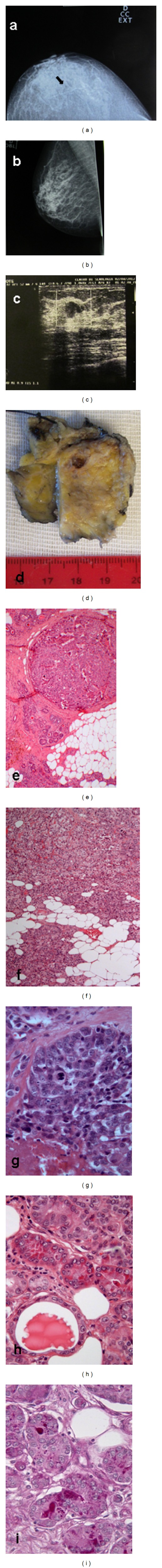
Craniocaudal (a) and oblique (b) view of mammography imaging showing radiopacity and thickening with microcalcifications (arrow). (c) Echographic study of the lesion showing, in the same area of (a) and (b), some nodular features. (d) Gross feature of the surgical specimen showing irregular whitish nodule of about 3 cm of diameter with a 8 mm small pseudocystic area. (e) Panoramic view showing nodular and pseudocystic arrangement of tumor (H/E, 100x). (f) Microglandular and microacinar features of tumour cells with small glandular structures arranged back to back and interspersed within fat or fibrous septa (H/E, 100x). (g) High power view of the solid features of the lesion showing high pleomorphic cells, mitotic activity, and necrosis (H/E, 400x). (h) High power view of microglandular structures showing colloid-like secretion in the central lumina and cytoplasmic eosinophilic granules (H/E, 400x). (i) High power view showing colloid-like secretion and cytoplasmic eosinophilic granules after PAS-diastase reaction (H/E, 400x).

Mammographic imaging (Figures 1(a) and 1(b)) showed a thickening with microcalcifications in the periareolar region of the right breast, while echographic study (Figure 1(c)) highlighted, in the same area, some nodular lesions.

A fine needle aspiration cytology was performed but it was unsatisfactory for diagnosis because of inadequate material.

The patient underwent a surgical biopsy with wide excision of the lesion: the specimen (Figure 1(d)) was a 5 × 5 × 2 cm mammary tissue with a 3 × 1 cm overlying skin ellipse. On cut section an irregular whitish nodule of about 3 cm of diameter was found with a 8 mm small pseudocyst area.

On microscopic examination the tumour showed a solid arrangement composed of cells with microglandular and microacinar features (Figures 1(e) and 1(f)).

The cyst-like area was composed of highly pleomorphic cells with high mitotic count and apoptotic bodies, arranged in solid nests with a central comedo-type necrosis (Figure 1(g)); in the microacinar areas glandular structures with central lumina (Figure 1(h)) interspersed within fat or fibrous septa could be seen. The central lumina contained an eosinophilic, colloid-like, PAS positive, and diastase-resistant secretory material (Figure 1(i)).

The cells appeared to have abundant eosinophilic cytoplasm and round nuclei with prominent nucleoli. At high power examination, the tumour cells focally showed a cytoplasm engulfed by eosinophilic granules (Figure 1(h)), appearing PAS positive and thus reminiscent of Paneth cells.

Microcalcifications were also observed within the tumour.

Glandular structures were devoid of myoepithelial basal layer such as evidenced by negative immunostaining for CD10, actin, and CK5/6.

**Figure 2:**
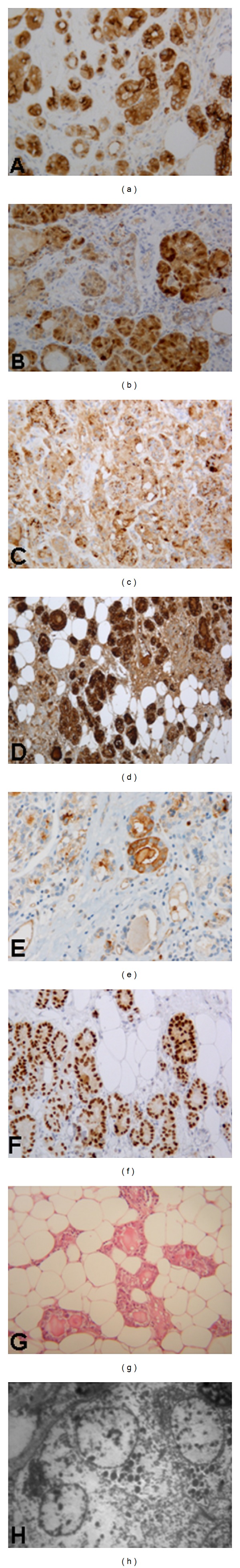
(a) Immunohistochemical assay for EMA (200x). (b) Immunohistochemical assay for S-100 (200x). (c) Immunohistochemical assay for *α*-1-antitrypsin (200x). (d) Immunohistochemical assay for lysozyme (200x). (e) Immunohistochemical assay for amylase (200x). (f) Immunohistochemical assay for progesterone receptor (200x). (g) Panoramic view of the section showing foci of microglandular adenosis at the periphery of the tumour (H/E, 200x). (h) Ultrastructural study showing variable sized electron dense cytoplasmatic granules.

Neoplastic cells showed immunoreaction for EMA (Figure 2(a)), CK7, E-cadherin, S100 (Figure 2(b)), *α*-1-antitrypsin (Figure 2(c)), lysozyme (Figure 2(d)), and amylase (Figure 2(e)). NSE, chromogranin, synaptophysin, androgen receptor, estrogen receptor, Her-2, TTF-1, and thyroglobulin were negative. GCDFP-15 resulted to be focally positive in the microacinar areas as well as progesterone receptor (Figure 2(f)). CD56 stained only the solid areas.

At the periphery of the tumor typical and atypical MGA was observed, it gradually merged into the ACC (Figure 2(g)).

Based on the morphological and immunohistochemical findings a diagnosis of salivary gland-like tumour of the breast with features of ACC arising in MGA was made.

An ultrastructural study was performed on formalin fixed paraffin embedded tissue: it showed variable sized electron dense cytoplasmatic granules (Figure 2(h)).

Since the tumour involved the surgical margins, surgeons performed a re-excision that showed some further focal areas of MGA. A sentinel node biopsy was also performed and the lymph node was negative for neoplastic deposits.

Moreover, the slides of the previous thyroid cancer were reviewed confirming a diagnosis of anaplastic thyroid cancer though were observed some areas with a differentiated follicular pattern containing a colloid-like eosinophilic secretion.

## 3. Discussion

ACC of the breast is a very rare subtype of mammary carcinoma and it belongs to the group of salivary gland-like tumors of the breast: a broad histological spectrum of breast tumors similar to those more commonly occurring in the salivary glands [2]; they represent approximately 2% of primary breast carcinomas [3].

Breast and salivary glands share embryologic similarities since both composed of tubuloacinar exocrine glands [2, 4].

ACC is a malignant tumor showing acinar cell differentiation with typical morphological and immunohistochemical findings and it was first described by Roncaroli et al. in 1996 [1].

To the best of our knowledge hitherto only 26 cases of ACC have been reported in the English literature [1, 4–18], among these we found only one case describing ACC arising in a MGA background [9].

Primary ACC of the breast presents in women between 35 and 80 years of age as a palpable lump, with the right side being more commonly affected. This special histological subtype is usually negative for estrogen receptors, progesterone receptors, and HER2 (so called “triple negative” cancers) [3]; however, recently, some authors reported a positivity for both estrogen and progesterone receptors in a case of ACC [15], as observed in the present case in which we found immunoreactivity for progesterone receptors.

Moreover, morphologically, a striking MGA, with typical and atypical features, gradually merging into ACC has been observed in our patient: so it is the second case of ACC arising on MGA to be reported in literature. In Table 1 we described clinical-pathological features of our case and of the previous one describing ACC arising on MGA.

MGA is a well-known benign breast lesion that has been reported in association with breast cancer: particularly Salarieh and Sneige reported association between breast carcinomas and MGA in up to 27% of cases [19]. Some authors found a more higher percentage of carcinoma arising in MGA, up to 64% of tumours of their study, probably due to bias selection [20].

Previous reports have shown that the pathogenesis of ACC is related to MGA [5, 9, 13, 20, 21].

Carcinoma arising in MGA can show several histological patterns like acinar, clear cells, matrix-producing, basal-like, or adenoid cystic [20].

In our case transition from areas of typical MGA into areas of atypical one until to merge, imperceptibly, into carcinoma is well evidenced also by immunohistochemistry, suggesting that ACC might origin from MGA.

It is imperative of obtaining free margins in case of MGA, as recently stated in the literature [16].

The present case was intriguing by the previous history of thyroid cancer and by the presence of colloid-like material in some glandular structures observed.

A legitimate question is whether it could be a thyroid carcinoma metastatic to the breast.

The slides of the previous thyroidectomy were reviewed, and a diagnosis of an anaplastic thyroid carcinoma was morphologically and immunohistochemical confirmed, even with the presence of areas with follicular pattern, resembling the microglandular features observed in the breast carcinoma.

Regarding the prognosis, ACC has been considered a rare tumor with good behavior; however axillary lymph nodes metastases have been found in 5 of the reported cases [1, 4, 5, 7, 14], while only one patient died of disease [2]; very recently a poor prognosis associated to ACC has been suggested [4].

The significance of a breast carcinoma with acinic cell differentiation remains unclear.

This is an extremely rare report of a primary ACC of the breast arising from a MGA.

Although it is rare, it should be considered in the differential diagnosis of breast cancer.

## Figures and Tables

**Table 1:** Literature review for acinic cell carcinoma of the breast arising on MGA background.

	Age/sex	Tumor size/side	N+	Surgery	Followup	MGA background	Solid growth pattern	Clear cells	Comedo necrosis	EMA	S-100	*α*-1-antitrypsin	Lysozyme/amylase	ER	PR	Her-2	GCDFP-15
Kahn et al.2003 [9]	56/F	22/L	0/18	RM + ALND	2/AW	Y	NO	NO	NO	+	+	NA	+/NA	NA	NA	NA	NA
Present case	58/F	30/R	0/1	BCS + SN	10 mo/AW	Y	Y	Y	Y	+	+	+	+/+	−	+	−	+/−

MGA: microglandular adenosis; N: nodes; ALND: axillary lymph node dissection; RM: radical mastectomy; BCS: breast conserving surgery; SN: sentinel node; AW: alive and well; Y: yes; NA: not available; R: right; L: left; F: female.
